# Iron Source and Medium pH Affect Nutrient Uptake and Pigment Content in *Petunia hybrida* ‘Madness Red’ Cultured In Vitro

**DOI:** 10.3390/ijms23168943

**Published:** 2022-08-11

**Authors:** Ge Guo, Jie Xiao, Byoung Ryong Jeong

**Affiliations:** 1 Department of Horticulture, Division of Applied Life Science (BK21 Four), Graduate School of Gyeongsang National University, Jinju 52828, Korea; 2 Institute of Agriculture and Life Science, Gyeongsang National University, Jinju 52828, Korea; 3 Research Institute of Life Science, Gyeongsang National University, Jinju 52828, Korea

**Keywords:** antioxidant system, chelate iron, chlorosis, iron chelate, nutrient absorption, *Petunia hybrida*, plant pigment

## Abstract

Deficiency or excess of iron (Fe) and improper medium pH will inhibit the growth and development of plants, reduce the transfer and utilization of energy from the root to the leaf, and affect the utilization efficiency of inorganic nutrients. The most common symptom of Fe deficiency in plants is chlorosis of the young leaves. In this study, the effects of the iron source, in combination with the medium pH, on plant growth and development, plant pigment synthesis, and nutrient uptake in a model plant *Petunia hybrida* cultured in vitro were investigated. Iron sulfate (FeSO_4_·7H_2_O) or iron chelated with ethylenediaminetetraacetic acid (Fe-EDTA) were supplemented to the MNS (a multipurpose nutrient solution) medium at a concentration of 2.78 mg·L^−1^ Fe, and the treatment without any Fe was used as the control. The pH of the agar-solidified medium was adjusted to either 4.70, 5.70, or 6.70 before autoclaving. The experiment was carried out in an environmentally controlled culture room with a temperature of 24 °C with 100 µmol·m^−2^·s^−1^ photosynthetic photon flux density (PPFD) supplied by white light emitting diodes (LEDs) during a photoperiod of 16 h a day, 18 °C for 8 h a day in the dark, and 70% relative humidity. Regardless of the Fe source including the control, the greatest number of leaves was observed at pH 4.70. However, the greatest lengths of the leaf and root were observed in the treatment with Fe-EDTA combined with pH 5.70. The contents of the chlorophyll, carotenoid, and anthocyanin decreased with increasing medium pH, and contents of these plant pigments were positively correlated with the leaf color. The highest soluble protein content and activities of APX and CAT were observed in the Fe-EDTA under pH 5.70. However, the GPX activity was the highest in the control under pH 4.70. In addition, the highest contents of ammonium (NH_4_^+^) and nitrate (NO_3_^−^) were measured in the FeSO_4_-4.7 and EDTA-5.7, respectively. More than that, the treatment of Fe-EDTA combined with pH 5.70 (EDTA-5.7) enhanced nutrient absorption, as proven by the highest tissue contents of P, K, Ca, Mg, Fe, and Mn. The genes’ ferric reduction oxidase 1 and 8 (*PhFRO1* and *PhFRO8*), iron-regulated transporter 1 (*PhIRT1*), nitrate transporter 2.5 (*PhNRT2.5*), and deoxyhypusine synthase (*PhDHS*) were expressed at the highest levels in this treatment as well. In the treatment of EDTA-5.7, the reduction and transport of chelated iron in *P. hybrida* leaves were enhanced, which also affected the transport of nitrate and catalyzed chlorophyll level in leaves. In conclusion, when the medium pH was adjusted to 5.70, supplementation of chelated Fe-EDTA was more conducive to promoting the growth and development of, and absorption of mineral nutrients by, the plant and the expression of related genes in the leaves.

## 1. Introduction

Iron (Fe) is the fourth-most-abundant nutrient on earth, and one of the most important nutrients for biological systems [1,2]. It is involved in the energy generation and utilization of plants and plays an important role in many redox reactions, and the absorption, distribution, and storage of Fe by plants are strictly regulated, so the transfer and absorption of Fe in leaves is the focus of the metabolism of the photosynthetic mechanisms [3]. Fe is primarily present in two forms: ferrous (Fe^2+^) and ferric (Fe^3+^). Fe^2+^ is more soluble and more easily oxidized, forming sediment with inorganic anions [4]. The solubility of Fe^3+^ decreases with increasing pH, mainly due to the lower solubility of hydroxide polymers, which in most cases cannot satisfy the needs of plants [5]. Since the concentration of free OH^–^ ions exceeds the Fe^3+^-OH formation threshold at neutral pH, maintaining a reduced pH environment and, thus, providing accessible Fe^2+^ for metabolic processes is critical [6]. Thus, the variable pH circumstances in a biological system necessitate multiple strategies for Fe–plant-species coordination [3]. Plants have devised two strategies to solubilize Fe. The reduction-based strategy, or so-called strategy I, is observed in most flowering plants. Graminaceous species display the chelation-based strategy, or strategy II [7]. The plants of strategy I take up Fe in roughly three steps: the roots release protons to increase the solubility of Fe^3+^, the Fe^3+^ is converted to Fe^2+^ by ferric chelate reductase (FCR), and then the Fe^2+^ is absorbed by the roots through the iron-regulated transporters (*IRT1*) [8]. The plant Fe-absorption system of strategy II synthesizes and secretes mugineic acid (MA) by S-adenosylmethionine (SAM) through an enzymatic reaction [9,10], and the enzymes involved in this synthesis mainly include nicotinamide synthase (NAS), nicotianamine aminotransferase (NAAT), and deoxymugineic acid synthase (DMAS) [11]. The Fe^3+^ in soils or media forms chelates with MA and is absorbed into cells through yellow stripe-like (YSL) transport.

In the case of Fe deficiency, the photosynthesis of plants is affected, as well as the absorption and accumulation of nutrients, and multiple signaling molecules, such as H_2_S, ethylene, and nitric oxide (NO), are involved to modulate the deficiency [12,13,14]. As a biologically active free radical, NO participates in and regulates the physiological functions of plants. NO is closely related to the process of Fe absorption, transport, and reoxidation. It can stimulate the Fe chelate reductase, significantly up-regulate the gene expression of ferric reduction oxidase 1 (*FRO1*) of Fe^3+^ and transporter *IRT1* of Fe^2+^, thereby increasing Fe absorption and homeostasis [15]. Fe is reduced to the ferrous form at the root surface and transferred to the leaf in a combined form via the xylem. This suggests that the encoded membrane transporter is also active in the aerial parts of the plant, even though MAs are predominantly found in the roots [4]. The acquisition in the plant of Fe and other micronutrients starts in the apoplast of the epidermal cells in roots [16]. The Fe is released into the xylem vessels on behalf of a transfer from symplasm to the apoplast [17,18]. Transporters of ferric–citrate, iron–nicotianamine, or other Fe complexes must moderate Fe absorption from xylem vessels, and photoreduction of the xylem carrying Fe carboxylate appears to play a key role in decreased Fe levels in the aerial parts [19]. The majority of Fe that reaches the leaves are from the Fe absorbed by the roots. The sink-source Fe distribution through the phloem is thought to provide the basis for Fe translocation towards the youngest leaves [20,21].

When plants are Fe-deficient, photosynthesis is affected, and chlorophyll cannot be produced normally; typical Fe-deficiency symptoms are the yellowing of young leaves [1]. In the tetrapyrrole biosynthesis pathway, Fe affects the development of coproporphyrin as well as the formation of chlorophyll [22,23,24]. Moreover, Liu et al. [25] also found that the expression of deoxyhypusine synthase (DHS) protein in *P. hybrida* was related to photosynthesis, and the chlorophyll level was significantly reduced in DHS-silenced leaves of *P. hybrida*, showing a chlorotic leaf phenotype. Moreover, Fe deficiency in plants also affects the production and metabolism of carotenoids and anthocyanins. They are synthesized in chloroplasts and play a vital role in the integrity of photosynthetic organs. There is also an inseparable relationship between the anthocyanins and the pH of the substrate [26]. The anthocyanin-production pathway is regulated by transcription factors, such as v-myb avian myeloblastosis viral oncogene homolog (MYB), basic helix-loop-helix (bHLH), etc. [27,28,29,30]. Most of the main biochemical reactions that participate in Fe metabolism are completed in the chloroplast, such that the chloroplast is the Fe pool in the plant cell. Fe deficiency will inhibit the conduction of photosynthetic electron chains in the chloroplast and cause optical damage [31].

More than 70% of the cations and anions absorbed by plants contain ammonium (NH_4_^+^) and nitrate (NO_3_^−^) [32], and the absorption and accumulation of these two nitrogen (N) sources affect the pH of the plant rhizosphere and apoplast [33], thus influencing the absorption and utilization of Fe. Excessive accumulation of NH_4_^+^ in plant cells results in toxicity and decreased photosynthesis, and symptoms such as hindered growth, chlorosis, etc., appear [34]. However, NO_3_^−^ accumulation in the rhizosphere increases the pH, reduces the Fe reductase activity, and inhibits Fe transport from root to shoot. Plants possess a regulatory system used for coordinating the uptake and distribution of these nutrients. In the cultivation process of *A. thaliana*, the nitrate and/or glucose supplemented to the medium has been shown to alter the levels of carbon (C) and N metabolic genes [35,36]. Therefore, in this study, the contents of NH_4_^+^ and NO_3_^−^, and the expression of transport genes were analyzed to explore the indirect effects of Fe and pH on the absorption of NH_4_^+^ and NO_3_^−^ in vitro. During the provision of different contents of NH_4_^+^ and NO_3_^−^, the uptake and transport of Fe in plants were affected. However, the effects on plant uptake and transport of nutrients in vitro were investigated when the same contents of NH_4_^+^ and NO_3_^−^ were provided, along with different Fe sources and pHs.

The bedding plant *P. hybrida* belongs to strategy I, has a short growth cycle, and is extensively used as a model plant of developmental biology [37]. *P. hybrida* is an Fe-inefficient plant that is sensitive to neutral and high pH [38] and responds by reducing the Fe uptake, which generates chlorosis and growth retardation [39]. Moreover, petunia is also very sensitive to the surrounding NH_4_^+^ and NO_3_^−^ [40,41]. In some previous studies, the effects of the Fe source and pH on the growth of shrub ornamental plants *Sorbus commixta* [42] and *Hydrangea macrophylla* [43], as well as the regulation of Fe-handling gene expressions, were studied. Moreover, in previous studies, more attention was paid to the effects of the NH_4_^+^ and NO_3_^−^ supply on the Fe absorption of plants, while studies seldom discussed the effects of different Fe sources and pH on plant ammonium and nitrate contents. Therefore, in this study, by providing different Fe sources and pH levels to *P. hybrida*, we investigate the effects on the growth characteristics, pigment contents, NH_4_^+^ and NO_3_^−^ contents, and their gene expressions.

## 2. Results

### 2.1. Plant Morphological and Growth Parameters

**Figure 1:**
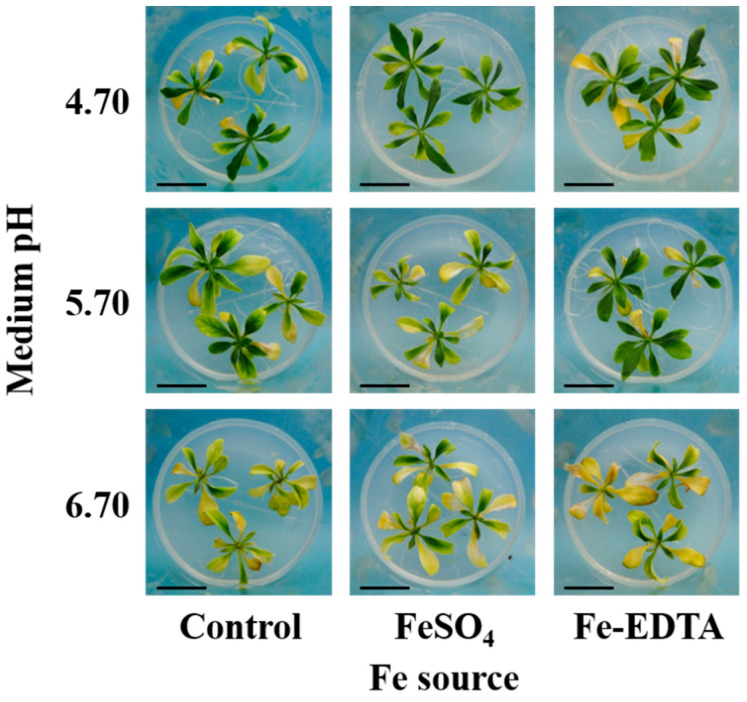
Photographs showing the growth and morphology of *P. hybrida* leaves as affected by the Fe source and medium pH after 10 days of culture in vitro. Bars indicate 3 cm.

The *P. hybrida* plants were cultured in a growth container for 10 days. The morphology of *P. hybrida* is shown in Figure 1 and Table 1. The morphological characteristics of *P. hybrida* were affected by the Fe source and pH level. In the treatment with high pH among these Fe sources, plants showed obvious chlorosis of leaves, which was the most serious at pH 6.70. The shoot length was the greatest in the control (no Fe) at pH 5.70 (6.3 mm) (control-5.7). The shoot length decreased with an increase in pH after supplying FeSO_4_ and Fe-EDTA. The greatest root length was observed in EDTA-5.7. The greatest shoot fresh weight (FW) was observed when the pH was 5.70 in the control. When the pH was 4.70 in FeSO_4_ (FeSO_4_-4.7) and Fe-EDTA (EDTA-4.7) treatments, the FW was greater than in other pH levels. The FW of roots was the greatest when the pH was 5.70, followed by pH 4.70 and 6.70. According to the results of the *F*-test in Table 1, the Fe source and pH affected the growth characteristics of the shoots and roots.

### 2.2. Leaf Color and Pigment Contents

**Figure 2:**
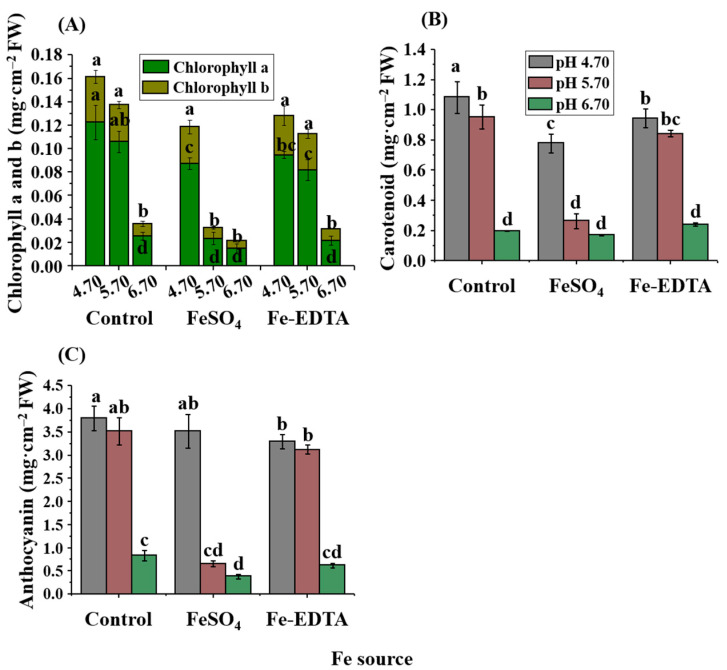
Effects of the Fe source and pH on the contents of total chlorophyll (**A**), carotenoid (**B**), and anthocyanin (**C**) in *P. hybrida* leaves after 10 days of culture in vitro. Different letters above the bars indicate significant differences according to the Duncan’s multiple range test at *p* ≤ 0.05. The standard errors (*n* = 3) are indicated by the vertical bars.

At the end of the experiment, the leaf color of three biological replicates of each treatment was identified by the color reader, and the coefficient differences of red (R), green (G), and blue (B) of each treatment in Table 2 were obtained according to the Munsell color system. Leaf color reflects the pigment contents of leaves. In the evaluation of the RGB chromatography, the RGB is a theory of light color mixing, where the value range of R, G, and B is between 0 to 255 [44]. The brighter the color of the leaves was observed to be, the greater the numerical value. When the pH was 6.70, the leaves were chlorotic. The same trend was also reflected in the pigment contents of leaves (Figure 2). When the pH of each Fe source was 4.70, the contents of chlorophyll, carotenoid, and anthocyanin were the highest among the three pH levels. At pH 6.70, these contents were the lowest.

According to Pearson’s correlation analysis results in Table 3, there is a positive correlation among the R value, the G value, and the B value, and there is also a positive correlation between the plant pigment contents in leaves. However, there is a negative correlation between each RGB factor and the plant pigment contents, thus resulting in smaller RGB values (deeper color) that correspond to higher pigment contents. Simultaneously, the values of the correlation were closer to −1 or 1, indicating greater significance of the correlation.

### 2.3. FCR Activity in Roots

**Figure 3:**
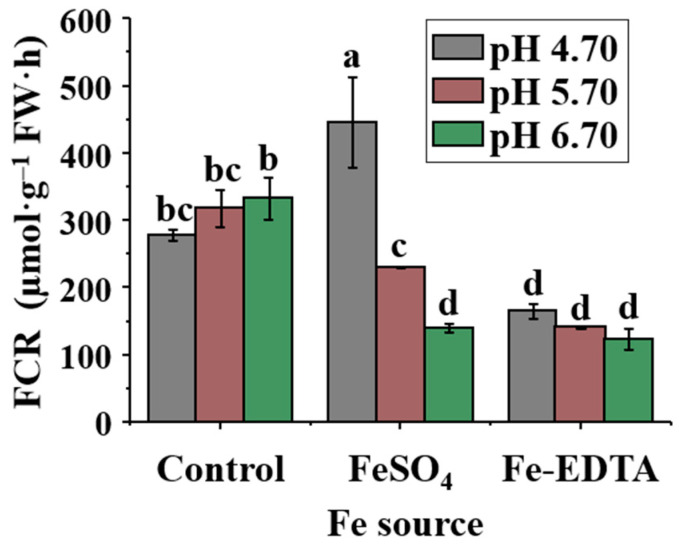
Effects of the Fe source and pH on FCR activity of *P. hybrida* after 10 days of culture in vitro. Different letters above the bars indicate significant differences according to the Duncan’s multiple range test at *p* ≤ 0.05. The standard errors (*n* = 3) are indicated by the vertical bars.

The FCR activity was affected by the Fe source and pH (Figure 3). The pH level in combination with FeSO_4_ significantly affected the FCR activities, and the FCR activity decreased with increasing pH, resulting in the highest FCR activity in FeSO_4_-4.7. However, the FCR activity was not affected by the pH in the control and in plants supplied with Fe-EDTA, although the FCR activities were significantly lower in treatments with Fe-EDTA than in the control.

### 2.4. Soluble Protein Contents and Antioxidant Enzyme Activities

**Figure 4:**
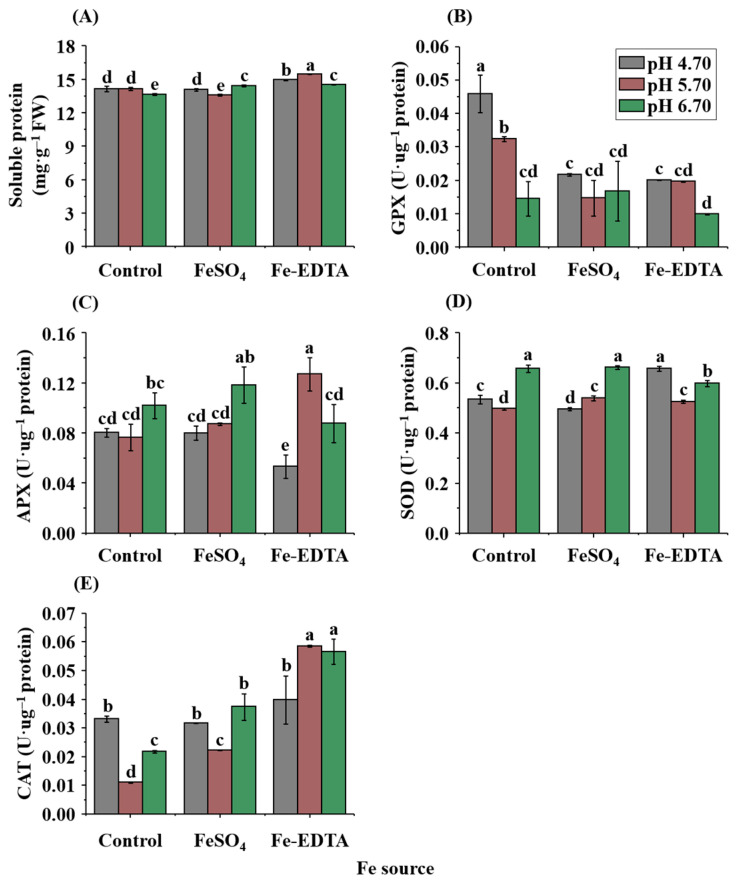
Effects of the Fe source and pH on the soluble protein content (**A**), activities of guaiacol peroxidase (GPX) (**B**), ascorbate peroxidase (APX) (**C**), catalase (CAT) (**D**), and superoxide dismutase (SOD) (**E**) in leaves of *P. hybrida* after 10 days of culture in vitro. Different letters above the bars indicate significant differences according to the Duncan’s multiple range test at *p* ≤ 0.05. The standard errors (*n* = 3) are indicated by the vertical bars.

The soluble protein content was significantly different among the treatments. The soluble protein content with Fe-EDTA treatments was generally higher, and when the pH was 5.70, the greatest soluble protein content (15.5 mg·g^−1^) was observed (Figure 4A). The antioxidant enzyme activities were significantly affected by the Fe source and pH. The maximum guaiacol peroxidase (GPX) activity was observed in control-4.7 (0.046 U·µg^−1^), while higher activities in the control group and the group with Fe-EDTA corresponded to a lower pH (Figure 4B). According to Figure 4C, the greatest ascorbate peroxidase (APX) activity was observed in EDTA-5.7 (0.127 U·µg^−1^), while in plants grown with FeSO_4_, the highest activity was observed in pH 6.70. Similar to the results of APX activity, the highest catalase (CAT) activity was observed in EDTA-5.7 (0.058 U·µg^−1^), as shown in Figure 4D. The greatest superoxide dismutase (SOD) activity was observed at pH 6.70 in the control and treatments with FeSO_4_ and at pH 4.70 with Fe-EDTA (Figure 4E).

### 2.5. NH_4_^+^ and NO_3_^−^ Contents

**Figure 5:**
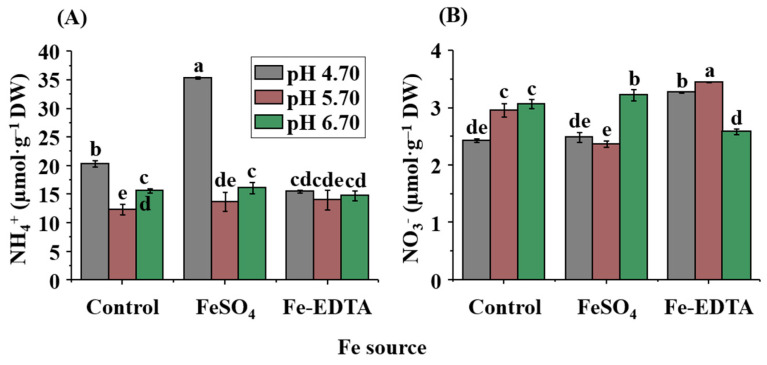
Effects of the Fe source and pH on the contents of NH_4_^+^ (**A**) and NO_3_^−^ (**B**) and in leaves of *P. hybrida* after 10 days of culture in vitro. Different letters above the bars indicate significant differences according to the Duncan’s multiple range test at *p* ≤ 0.05. The standard errors (*n* = 3) are indicated by the vertical bars.

According to Figure 5, the Fe source and pH had significant effects on the contents of NH_4_^+^ and NO_3_^−^ in *P. hybrida* leaves. In the control and FeSO_4_ groups, the highest content of NO_3_^−^ was measured at pH 6.70. For Fe-EDTA, the highest content of NO_3_^−^ was observed at pH 5.70. The lowest NO_3_^−^ content was observed at pH 6.70 with Fe-EDTA, whereas the control and FeSO_4_ had the greatest NO_3_^−^ content at pH 6.70. In Figure 5A, the variation of the NH_4_^+^ content was influenced by the medium pH. Therein, FeSO_4_-4.7 had the highest content (35.34 µmol·g^−1^) among all treatments, and the lowest content was measured in control-5.7 (12.36 µmol·g^−1^).

### 2.6. Contents of Macronutrients and Micronutrients in Leaves

As shown in Table 4, the Fe source and pH level directly affected the uptake of nutrients in *P. hybrida*. In the control and FeSO_4_, lower pH levels corresponded to higher Fe contents. On the other hand, the Fe content was the greatest at pH 5.70 in the Fe-EDTA and the lowest at pH 4.70. The efficient Fe uptake also promoted the uptake of other nutrients. In EDTA-5.7, which contained the highest Fe content, the contents of micronutrient manganese (Mn) and macronutrients phosphorus (P), potassium (K), calcium (Ca), and magnesium (Mg) were also the greatest.

### 2.7. Correlation Analysis of Pigments with Fe Content, Growth Parameters, and Nutrient Contents in Leaves

Table 5 and Table 6 show the correlation among the contents of three pigments, growth parameters of shoots, and 12 nutrient contents. The Fe content had a positive correlation with the contents of carotenoid, and had a significant positive correlation with chlorophyll and anthocyanin (Table 5). However, there was no significant correlation between Fe content and the number of leaves or between the length and FW of shoots (Table 6). There was a very significant negative correlation between shoot DW and Fe content. In addition, there is also a very significant negative correlation between DW and contents of Zn, Cu, Mn, Ca, Mg, and K, indicating that the higher contents of these nutrients in shoots corresponds to a lower DW and a higher water content. The contents of Mn, Ca, K, and P had a very significant positive correlation with the number of leaves, indicating that higher contents corresponded to a greater number of leaves. The Fe content had a positive correlation with the contents of other nutrients except sulfur (S), and had a very significant positive correlation with contents of Cu, Mn, Ca, Mg, K and P. In the correlation results between NH_4_^+^ and NO_3_^−^, NH_4_^+^ was significantly negatively correlated with NO_3_^−^, while both were not significantly positively correlated with the Fe content. This indicated that there were no significant direct relationships between the Fe content and the NH_4_^+^ and NO_3_^−^ contents in *P. hybrida* leaves. The correlation values were closer to −1 or 1, indicating a greater significance of the correlation.

### 2.8. Expressions of Fe, Pigments, and N Transporter Genes

**Figure 6:**
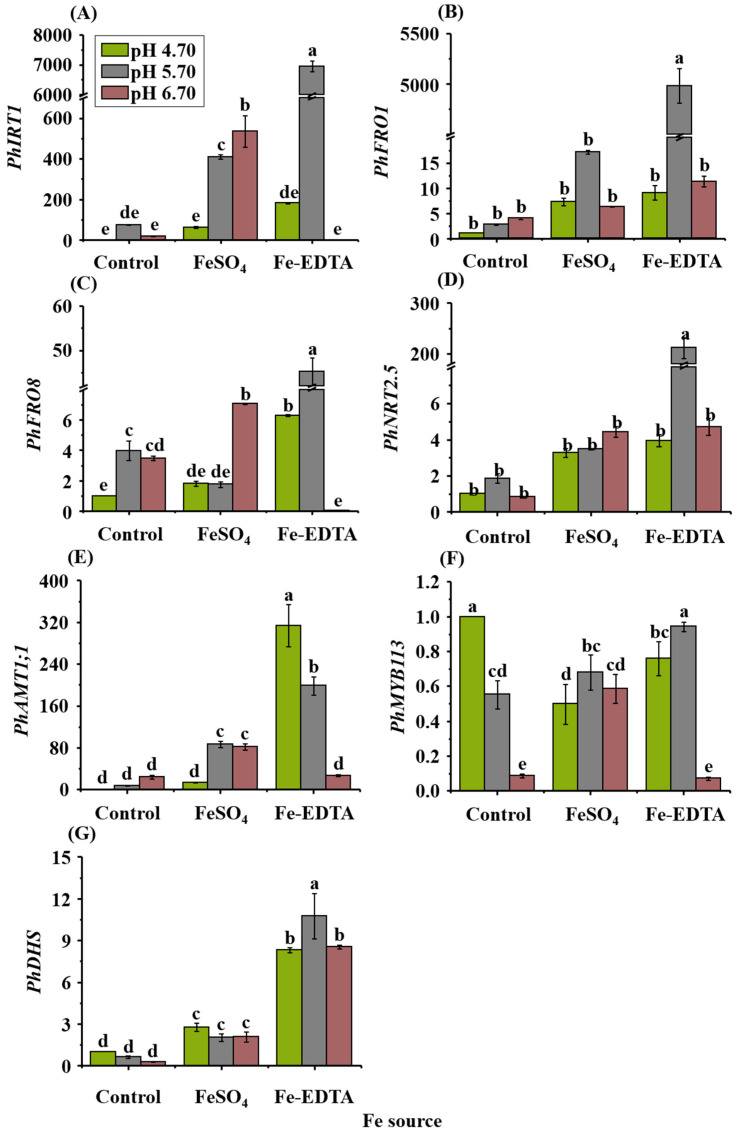
The gene expression levels in *P. hybrida* as affected by the Fe source and medium pH after 10 days of cultivation in vitro. Fe-regulated transporter, *PhIRT1* (**A**); ferric reduction oxidase, *PhFRO1* (**B**) and *PhFRO8* (**C**); ammonium transporter, *PhNRT2.5* (**D**); nitrate transporter, *PhAMT1;1* (**E**); myb domain protein, *PhMYB113* (**F**); and deoxyhypusine synthase, *PhDHS* (**G**). The relative transcription level of each gene was analyzed by quantitative real-time PCR (qRT-PCR). The expression of genes was normalized to that of *PhActin*. Different letters above the bars indicate significant differences according to the Duncan’s multiple range test at *p* ≤ 0.05. The standard errors (*n* = 3) are indicated by the vertical bars.

The relative expression levels of genes were significantly different according to the treatments (Figure 6). The expression levels of one gene related to Fe-regulated transporter (*PhIRT1*) (Figure 6A), two genes related to ferric reduction oxidase (*PhFRO1* and *PhFRO8*) (Figure 6B,C), one gene related to ammonium (*PhNRT2.5*) (Figure 6D) and one gene related to nitrate transport (*PhAMT1;1*) (Figure 6E), one gene related to myb domain protein (*PhMYB113*) (Figure 6F), and one gene related to deoxyhypusine synthase (*PhDHS*) (Figure 6G) were investigated in this study. All the highest relative expression of gene levels were observed in the treatment with Fe-EDTA, except for *PhAMT1;1* and *PhMYB113*. The highest relative expression levels of *PhAMT1;1* were observed in EDTA-4.7. The highest relative expression levels of *PhFRO1*, *PhFRO8*, *PhIRT1*, *PhNRT2.5*, and *PhDHS* were observed in EDTA-5.7. However, the highest relative expression level of *PhMYB113* was observed in control-4.7, followed by EDTA-5.7.

## 3. Discussion

Higher plants mainly uptake Fe by the root system. On account of a strong demand for Fe, the photosynthetic system will be established and manipulated in the chloroplast of the photosynthetically active tissue, and Fe is transported from the roots to leaves. Petunia is an Fe-inefficient plant that is sensitive to an environment of neutral or high pH, which would result in reduced Fe uptake, followed by appearance of chlorosis and growth inhibition [45,46]. This was also confirmed in this study (Figure 1), as leaves of petunia showed obvious chlorosis at high pHs. The Fe homeostasis in leaves depends on the binding of Fe to cofactors of oxidoreductases, and the developmental system of plants also determines the direction of the Fe distribution in leaf cells [3]. In this study, the effects of the Fe source and different medium pH on the plant growth and development of *P. hybrida* were obvious. According to the growth parameters in *P. hybrida* (Table 1), the plants with the highest number of leaves were observed at pH 4.70 regardless of the Fe source, including the control group. The pH affects the substance solubility and nutrient availability for plants. It is also confirmed in this study (Table 6) that the number of leaves has a significant positive correlation with the contents of Mn, Ca, K, and P in leaves. At the same time, it was also found that there was a significant regularity between the pH of the medium and the number of leaves and the content of these elements (Table 1 and Table 4). Among the iron sources, high pH corresponds to a lesser number of leaves and the content of these nutrient. H^+^ as a cation will exchange positions while competing with other cations. The metal availability is often inhibited, and micronutrient deficiencies are generated for plants in alkaline soils [47]. Moreover, the solubility of Fe under different pH levels also changes. In neutral pH media, Fe exists in the form of ferric Fe and most of the Fe^3+^-OH complexes are cross-linked into insoluble ferrihydrite polymers, therefore, the Fe ion content in the medium is extremely low [48]. The solubility of FeSO_4_ is higher at lower pH (below 5.5), and the solubility of FeSO_4_ decreases with the increase in the pH [49]. Fe-EDTA, a chelated Fe, is unstable at high pH; it is relatively stable at a pH lower than 6.0, and highly unstable when at a pH higher than 6.5. That explains why in Table 1, when the Fe source was Fe-EDTA, the leaf length and root length of *P. hybrida* were the greatest at pH 5.70.

In strategy I plants, reduction of Fe chelates is carried out by FCR, and plants are more likely to reduce Fe with weak Fe^3+^-chelates. In some Fe-sensitive species (such as *Vaccinium* spp. and *Annona glabra*), low FCR activity was found in acidic soils with high organic matter and Fe^2+^, where these conditions are more favorable for Fe uptake [50]. Following the FCR-mediated reduction, Fe^2+^ is transported through the plasma membrane into the roots [51]. Strategy I plants upregulate the expression of FCR enzymes when the Fe content of the medium is limited, while increasing the pH level will enhance the FCR activity [52,53], which was similar to the findings in Figure 3. The FCR activity was the highest in control-6.7 (pH is greater than 6.5) among the three pH levels in the control, because the Fe level in the medium affects the root response mechanisms to improve the Fe uptake [54]. The excess Fe^2+^ produced by FCR is re-oxidized by the electron acceptor, but the chelating agent can be re-oxidized by the catalysis of Fe^3+^-chelate [55]. Furthermore, the Fe availability affects the natural distribution of species and limits the growth of important commercial crops. To prevent the potential high Fe toxicity to plants [56], plants have evolved a series of protective mechanisms that bind Fe and proteins. Plants will reduce Fe uptake by reducing the FCR activity when the Fe content in the medium is excessive; for example, in *Spinacia oleracea* and *Brassica oleracea*, the FCR activity of roots decrease with increasing Fe content [57]. The Fe content in the Fe-EDTA was high on account of Fe-EDTA reducing Fe to Fe^2+^ by the reaction of metal charge transfer (Table 4) [58,59]. Therefore, the Fe-EDTA group showed a lower overall FCR activity.

Among the extensive range of metal transporters in plants, NRAMP (natural resistant-associated macrophage protein), YSL (yellow stripe-like), and ZIP (zrt- and irt-related protein) families are thought to participate in the Fe transport [60]. As a member of the ZIP family, *IRT1* is capable of compensating for some defects in the Fe uptake, and IRT2 is also a homologue of the ZIP family [61]. *IRT1* and *IRT2* uptake ferrous Fe from the medium in Fe deficiency, and its gene expression is induced in the roots as a part of the Fe-deficiency response [62,63]. In Fe-deficient *A. thaliana*, Fe and other metals are absorbed by *AtIRT1* from the medium to regulate Fe homeostasis, and *AtIRT1* is highly expressed in the epidermal region of the roots [64] (in strategy I plants, the expression of proton pumping, Fe chelate reductase, and *IRT1* are all significantly increased when Fe is deficient) [65]. In the EDTA-5.7 treatment in this study, the expression level of *PhIRT1* in *P. hybrida* leaves was 6947 times higher than that in the control (Figure 6A), which may be because when the ligand inside plants is binding to metal cations, the high mobility relative to the lower affinity of the chelated Fe is at a negative location of cell membranes and vessels [66]. It was also found that in *Zea mays*, chelating agents such as EDTA or DTPA will increase the Fe transport in leaves. Brian et al. [67] discovered that the expression of *FRO1* was low in the Fe-rich leaves of *Pisum sativum*, while the expression of *FRO1* was increased in the Fe-deficient leaves [68]. The highest *PhFRO1* expression and Fe content were observed in EDTA-5.7 in this study (Figure 6B); before uptake of Fe^3+^ by leaf cells, *FRO1* participated in the process of composite reduction of Fe^3+^ to Fe^2+^ [67,69]. *FRO8*, which is also a chelate Fe reductase, exists in the mitochondria of cells, mainly acts on Fe reduction during shoot senescence [70], and also participates in the reduction of Fe^3+^ to Fe^2+^. Simultaneously, the highest expression of *PhFRO8* was observed in EDTA-5.7 (Figure 6C).

The plant pigment contents in leaves are closely related to the physiological function of leaves, which can provide a valuable insight into the physiological functions of leaves. According to the contents of total chlorophyll (chlorophyll a and b), carotenoids, and anthocyanins in Figure 2, the contents of these photosynthetic pigments and secondary metabolites were influenced by the Fe source and medium pH. However, the effects of the medium pH were stronger than that of the Fe source. As shown In Figure 1 of this study, when the pH was 6.70, the leaves were chlorotic. The same trend was also observed in the pigment contents in leaves. This is similar to the results of Smith et al. [38,39], where when the pH of the substrate was increased from 5.0 to 6.0, the contents of the total chlorophyll and carotenoids in leaves of *P. hybrida* ‘Priscilla’ decreased, chlorosis appeared after 2 weeks, and, when the pH of the medium was raised to 7.0, the contents of total chlorophyll and carotenoid decreased significantly. Chlorosis of young leaves in general is the first visual phenomenon of Fe deficiency. It is associated not only with the damage of chlorophyll but also with changes in the expression and assembly of other components of the photosynthetic organ [71]. In photosynthetic cells, approximately 80% of the Fe is a key determinant in the formation of prosthetic groups in enzymes [72]; the Fe content plays a role in a variety of vital plant activities such as chlorophyll production, electron transport between PSI and PSII, and maintenance of the structure and function of the chloroplast [73].

The function of chloroplasts depends on the plastid and nuclear genomes of proteins encoded, and a normal development of chloroplasts is associated with the leaf color [74,75]. The DHS proteins localized in the cytoplasm and nucleus affect the chlorophyll levels in leaves, and in *Solanum lycopersicum* and *Nicotiana tabacum* L., the formation of deoxyhypusine residues is catalyzed by DHS, which plays a role in chloroplast development [76]. Liu et al. [25] observed that in *DHS*-silenced leaves in petunia, the chloroplast development had abnormal and reduced chlorophyll levels and activity of photosystem II. However, in our study, the highest expression of the *DHS* gene appeared in EDTA-5.7, where the chlorophyll content and chlorosis state were about the median among all the treatments; it is, therefore, speculated that the DHS expression is more related to the pH. Fe reacts to the porphyrin structure of chlorophyll, the main component of the chloroplast. A famous function of the cytochrome is electron transfer, and cytochrome oxidase is involved in the last step of the respiratory chain [77]. The ratio of condensation for glycine and succinyl-CoA synthesis of ALA (delta-aminolevulinic acid) is reduced when plants are Fe-deficient. At that time, the photosynthetic system remains intact, but the number of photosynthetic units decrease [78]. Nenoval et al. [79] found that excess Fe can increase the pigment contents in pea leaves by at least 28%. The relationship between the chlorophyll content and spectral index is also affected by other pigments [26]. The anthocyanin content tends to be higher in young leaves with low photosynthetic rates [80], and metal ions and pH can alter the anthocyanin color in vitro but only play a minor role in vivo. The biosynthesis of anthocyanins is mainly regulated by several transcription factors in the R2R3-MYB family, of which *MYB113* mainly regulates the anthocyanin expression in mature leaves [81]. Several research data indicated that anthocyanin synthesis is enhanced when the *MYB113* gene is overexpressed in *A. thaliana* [30]. It was confirmed in our study that the control-4.7 treatment with the highest expression of *PhMYB113* (Figure 6F) also resulted in the greatest anthocyanin content (Figure 2C), indicating that the *PhMYB113* expression of the control group corresponded to the anthocyanin level. However, in treatment groups with FeSO_4_ and EDTA, the expression of *PhMYB113* was the highest at pH 5.70. Since nutrients affect the synthesis of anthocyanins and also activate or inhibit the expression of transcription factors [82], it is speculated that the Fe source and pH affect the expression of *PhMYB113.* In vegetative tissues, anthocyanins are considered important photosynthetic antioxidants [83,84]. When plants are deficient in anthocyanins, photoprotection is achieved through an alternative mechanism [85]. Therefore, the change in the anthocyanin content often also represents the diversity of antioxidants [86]. In the antioxidant processes of plants, H_2_O_2_ is oxidized by SOD and then scavenged by GPX, POD, and CAT. Under the influence of Fe and ROS, the activities of GPX, POD, and POD change accordingly [87]. As shown in Figure 2, the pH affects Fe absorption, resulting in reduced anthocyanin content with Fe deficiency.

Several steps of biosynthesis are dependent on Fe. Fe-deficiency chlorosis severely affects crop quality and production. Meanwhile, chlorosis caused by Fe deficiency in a high pH environment is generally ameliorated by other approaches, such as acidification of the medium or the use of amino-based fertilizers, to lower the pH to achieve higher solubility of microelements [45,88]. At the same time, the results of other research indicate that the correlation between the Fe concentration and chlorophyll content is not always the same, as in the case of chlorosis caused by Fe deficiency, and the Fe concentration in chlorotic leaves may be higher than that in non-chlorotic leaves. When the pH is too high, Fe can be fixed in the free space of leaves [89]. The Fe source and pH not only affect the plant pigment content but also the macronutrient and micronutrient contents [90]. Floral crops cultivated under soilless conditions are commonly cultivated with a medium pH range, usually controlled between 5.6 and 6.2. In the leaves of plants, the availability of nutrients correlates with the water solubility of the applied compound [66]. The solubility of nutrients is controlled by the pH. When the pH increases, the solubility of the microelements in the medium decreases, and oxyphilous plants appear as a nutrient-deficiency symptom. Meanwhile, when Fe-efficient plants such as *Pelargonium hortorum* and *Tagetes erecta* L. are grown in a low-pH medium with high solubility of Fe and Mn, elemental poisoning occurs, thereby generating necrosis in plants [45]. The high pH of the medium also restricts Mn uptake, and the symptoms of Mn deficiency are similar to those of Fe deficiency [91]. As shown in Table 4, in the control and FeSO_4_ groups, low Mn content corresponded to a high–medium pH. According to the results of Abadia et al. [92], when the leaves of *Prunus persica* L. appear to be chlorotic, the content of K ions increases, while the content of Ca ions in the leaves decreases, thereby the value of K/Ca is increased. However, as shown in Figure 1 and Table 4, chlorosis of leaves was obvious with the increase in the pH, and the content of K and Ca ions also decreased, especially in the control and FeSO_4_ groups. It has been reported that the reduction of chlorophyll is not always due to Fe deficiency but may also be related to the dilution effect of leaves, and the appearance of chlorosis is not necessarily directly related to the nutrient contents.

In addition, N is also an important mineral nutrient for crops, and an important factor for plant growth and development [93]. Some important organic compounds in plants are inseparable from N, such as proteins, chlorophyll, alkaloids, etc. [41]. N is mainly dissolved in the form of NH_4_^+^ and NO_3_^−^, then absorbed by the roots of plants [94]. A large proportion of NO_3_^−^ and a fraction of NH_4_^+^ are directly transferred to the leaves, and the remainder is assimilated [94,95]. The absorption of NO_3_^−^ and NH_4_^+^ significantly affected the pH of the rhizosphere and apoplast, thereby affecting the absorption and utilization of Fe by plants [96]. As shown in Figure 5 and Table 6, the nutrient contents interact with each other in *P. hybrida* leaves. In the control group under Fe deficiency, lower pH corresponded to lower NO_3_^−^ contents, because the increase in pH increased the NO_3_^−^ content, while reducing the activity of Fe^3+^ reductase, thereby inhibiting the transport of Fe from roots to shoots [97,98]. In the treatment with FeSO_4_ or chelated iron EDTA, due to supplementation of Fe^2+^ or Fe^3+^, the interaction effect and absorption by leaves of nutrients were changed. The N uptake of plants is mainly determined by specific genes, such as the nitrate transporter *NRTs* and ammonium transporter *AMTs* [99]. The regulation of the *NRT* and *AMT* expressions are affected by N metabolites, light, pH, sucrose, etc. [100,101]. Among them, NRT2.5 is a high-affinity nitrate transporter that is localized in the plasma membrane and mainly expressed in the epidermis of root hairs and leaves [102]. As shown in Figure 6D, in the control group, the expression level of *PhNRT2.5* was lower than that in the FeSO_4_ and EDTA groups, and the highest expression level was observed in EDTA-5.7. In addition, the overexpression of *AMT1* will increase the absorption of ammonium by plants, but, with sufficient ammonium content, *AMT1* will reduce the biomass of the aboveground parts in order to reduce ammonium toxicity [94]. As shown in Figure 6E, the expression of *PhAMT1;1* in the control group was the lowest and was affected by the pH, with high pH corresponding to high expression. The highest expression of *PhAMT1;1* was observed in the EDTA group, and the expression decreased with increasing pH. Therefore, we judged that under the synergistic effects of Fe and pH, the uptake and contents of NO_3_^−^ and NH_4_^+^ were affected in *P. hybrida* leaves.

## 4. Materials and Methods

### 4.1. Seed Materials and Sterilization

The bedding plant seeds of *Petunia hybrida* ‘Madness Red’ were purchased from PanAmerican Seeds (West Chicago, IL, USA). The seeds were washed with running tap water for 1 h to wash off the coating, then put into glass beakers with 0.3% (*v*/*v*) Tween 20 solution and stirred for 3 h using a blender. The seeds were subsequently rinsed with sterilized distilled water (SDW) 7 times. The seeds were then transferred to a sterilized container, and the surfaces of the seeds were sterilized with 70% (*v*/*v*) ethanol for 1 min and 0.5% (*v*/*v*) sodium hypochlorite (NaClO) for 5 min. Finally, the seeds were rinsed with SDW 5 times before culture.

### 4.2. Seed Germination and Culture Conditions

The surface-sterilized seeds were cultured in a multipurpose nutrient solution (MNS) medium for germination [103]. The medium contained 3.0% (*w*/*v*) sucrose and 0.80% (*w*/*v*) agar, and the pH was adjusted to 5.80. Then, 50 mL of the medium was dispensed to each container (Cat. No. Gaooze-0811C, Korea Scientific Technique Industry, Suwon, Korea) before being sterilized in an autoclave at 121 °C for 15 min. A total of 15 seeds were sown in each container and placed in the culture room with a temperature of 24 °C and relative humidity of 70%, and a 16 h photoperiod, provided at 100 µmol·m^−2^·s^−1^ photosynthetic photon flux density (PPFD) by white fluorescent light (40 W tubes, Philips, Eindhoven, The Netherlands).

### 4.3. Experimental Design of the Fe and pH Treatments

The seeds of *P. hybrida* were germinated and cultured in the MNS medium for 15 days, after which the plantlets were transplanted to the media with the Fe treatments. The composition was (in mg·L^−1^): Ca(NO_3_)_2_·4H_2_O 436.6, KNO_3_ 232.3, KH_2_PO_4_ 272.0, MgSO_4_·7H_2_O 209.1, NH_4_NO_3_ 80.0, K_2_SO_4_ 17.4, H_3_BO_3_ 1.40, CuSO_4_·5H_2_O 0.20, MnSO_4_·4H_2_O 2.10, Na_2_MoO_4_·2H_2_O 0.12, and ZnSO_4_·7H_2_O 0.80, containing 3.0% (*w*/*v*) sucrose and 0.80% (*w*/*v*) agar. The iron sulfate (FeSO_4_·7H_2_O) or iron chelated with ethylenediaminetetraacetic acid (Fe-EDTA) were used as the Fe sources, according to the protocol of Xiao et al. [42] with a final Fe content of 27.8 mg·L^−1^ in the MNS medium, and the treatment without Fe was used as the control. Before autoclaving, the pH of the medium was adjusted to 4.70, 5.70, or 6.70 with 1 M NaOH or HCl. Each treatment was repeated three times.

### 4.4. Measurements of the Growth Parameters

After 10 days, the length of the shoots and roots were examined. The FW of the shoots and roots was measured with a precision electronic balance (NTRIS224I-1S, Sartorius, Gottingen, Germany). Before the dry weights (DW) were measured, the shoot and root samples were dried in a forced convection oven (AAH12245K, Jeio Tech. Co., Ltd. Daejeon, Korea) at 60 °C for 72 h. The leaf-color data were collected with a color reader (CR-11, Konica Minolta Co., Ltd. Osaka, Japan). The obtained Munsell chromatographic data were converted into RGB chromatographic data using an online tool (https://pteromys.melonisland.net/munsell/) (accessed on 7 February 2022), and after obtaining the average of at least 3 biological replicates, the RGB chromatographic data were converted into an exclusive serial number of HEX chromatographic data online (https://www.rgbtohex.net/) (accessed on 7 February 2022).

### 4.5. Contents of Chlorophyll, Carotenoid, and Anthocyanin

The extraction solution was formed overnight using a 1 cm^2^ area of the leaf tissue soaked in 80% (*v*/*v*) acetone. Then, a UV spectrophotometer (Libra S22, Biochrom Ltd., Cambridge, UK) was used to evaluate the absorbances of the extraction solution at A_470nm_, A_645nm_, and A_662nm_. The contents of chlorophyll a, b, and carotenoid were measured according to the protocol of Sukran et al. [104]. The total anthocyanin was also evaluated using a UV spectrophotometer, according to the protocol of Pirie and Mullins [105].

### 4.6. Determination of the FCR Activity

The root FCR activity was measured based on the methods of Biengait et al. [106] and Chaney et al. [107]. The fresh roots from each treatment were washed clean of the residual solid medium, immediately put into the saturated stationary CaSO_4_ solution for 5 min, rinsed with SDW 2–3 times, wiped to remove the surface moisture, and put into a 15 mL FCR reaction solution [0.1 mM Fe-EDTA and 0.4 mM 2,2′-Dipyridyl, pH 5.3]. A solution sample without roots was used as the control, and all samples were incubated in the dark at 24 °C for 1 h. The absorbance of the reaction solution at A_520nm_ [1 mM Fe(dipyridyl)_3_ at A_520nm_ = 8.650] was measured and calculated with a UV spectrophotometer.

### 4.7. Soluble Protein Content and Antioxidant Enzyme Activity Assays

After 10 days, leaves of *P. hybrida* were separated from plantlets and frozen in liquid nitrogen immediately. The leaf tissues from different treatments were ground into a fine powder with liquid nitrogen and stored at −80 °C until the next analysis. The activities of antioxidant enzymes guaiacol peroxidase (GPX), catalase (CAT), ascorbate peroxidase (APX), and superoxide peroxidase (SOD) were measured according to Manivannan et al. [108]. Then, 0.10 g fine powder samples were homogenized with 100 mM phosphate-buffered saline (PBS) solution for the GPX assay and homogenized with 50 mM PBS solution to measure the activities of CAT, APX, and SOD. A 50 mM PBS solution containing 1 mM ethylenediaminetetraacetic acid (EDTA), 1 Mm polyvinylpyrrolidone (PVP), and 0.05% triton-X at pH 7.0 was used. The mixtures were centrifuged at 4 °C for 20 min at 13,000 rpm, then the supernatants were transferred to a new 1.5 mL microtube to analyze the soluble protein contents and activities of the antioxidant enzymes. The unit·µg^−1^ was used to represent the specific enzyme activity. The enzymes that induced a 0.01 per minute increase was determined as one unit of GPX activity. The enzymes required to reduce 1 µM of H_2_O_2_ for each minute was defined as one unit of CAT activity. The enzyme activity catalyzing the oxidation of 1 µM ascorbic acid each minute was defined as one unit of APX activity. The quantity that causes a 50% inhibition of the initial rate of nitro blue tetrazolium (NBT) degradation was determined as one unit of SOD activity.

### 4.8. Contents of NH_4_^+^ and NO_3_^−^

The NH_4_^+^ and NO_3_^−^ concentrations in leaves were analyzed based on the methods of Bräutigam et al. [109] and Huang et al. [110]. The NH_4_^+^ concentration was analyzed by a colorimetric method based on the Berthelot reaction [109]. The 0.10 g fine powder of frozen leaf tissues was homogenized with an extraction solution containing 1 mL 0.1 M of HCl and 500 µL of chloroform and shaken at 4 °C for 15 min and then centrifuged for 5 min at 12,000 rpm (4 °C). Then, the supernatants were transferred to a new microtube containing 50 mg of activated charcoal and then centrifuged for 5 min at 12,000 rpm (4 °C) after homogenization. The supernatants were analyzed for the NH_4_^+^ concentration with a UV-spectrophotometer at A_620nm_. For the NO_3_^−^ concentration, 0.10 g fine powder of frozen leaf tissues was extracted with 1 mL DW (deionized water) and incubated at 45 °C for 1 h. After centrifugation for 15 min at 5000 rpm (20 °C), 0.2 mL of the supernatant was transferred to a 30 mL glass tube and mixed with 0.8 mL 5.0% (*w*/*v*) salicylic acid in concentrated sulfuric acid. The solution was incubated for 20 min, and 19 mL of 2.0 M sodium hydroxide was then added to raise the pH of the solution to more than 12. After the solution was cooled to room temperature, the concentrations of NO_3_^−^ were analyzed with a UV-spectrophotometer at A_410nm_.

### 4.9. Macronutrient and Micronutrient Contents

The macronutrient and micronutrient contents were analyzed based on the methods of Zhang and Dotson [111]. The dry weight of the leaf samples was measured, and the leaves were then ashed for 4 h in a Nabertherm muffle furnace (Model LV 5/11/B180, Lilienthal, Breman, Germany) at 525 °C. The ashes of the leaves were dissolved in 1 mL of 25% HCl (hydrochloric acid), then dissolved in 2 mL of hot distilled water, and 3 mL of normal temperature distilled water was added. The total mixture (6 mL) was filtered through a quantitative filter paper (Cat no. 02005110, Hyundai Micro Co., Ltd., Seoul, Korea), and the macronutrient and micronutrient contents of mixture were analyzed with an inductively coupled plasma (ICP) spectrometer (Optima 4300DV/5300DV, Perkin Elmer, Baesweiler, Germany). Three biological replicates were performed for each treatment.

### 4.10. Quantitative RT-PCR Analysis

The RNA of the leaf tissues was extracted with the TRIzol Reagent (Cat no. 15596026, Invitrogen, Carlsbad, CA, USA) in accordance with the standard protocols of the manufacturer. Briefly, 0.1 g frozen powder of leaf tissues was ground with 1.0 mL TRNzol to homogenize, and the homogenate was transferred to a 1.5 mL tube (RNA-free), incubated for 10 min at room temperature, then centrifuged at 4 °C, 12,000 rpm for 11 min. The supernatant (0.8 mL) was transferred to a new 1.5 mL tube, to which 0.2 mL chloroform was added, mixed thoroughly, and centrifuged at 4 °C, 12,000 rpm for 11 min. Next, 0.4 mL of the supernatant was transferred to a new 1.5 mL tube, and an equal volume of isopropanol was added, then the mixture was inverted several times to mix and incubated at −20 °C. After 3 h of incubation, these samples were centrifuged at 4 °C with 12,000 rpm for 11 min. The supernatant was discarded with a pipette, 0.9 mL 75% (*v*/*v*) ethanol was added and mixed well with the pellet at 4 °C, 12,000 rpm for 11 min, and the process was repeated twice. Finally, the pellet was dried for 10 min at room temperature, dissolved with 20 µL ddH_2_O (double distilled water), then kept at −80 °C for further work.

A NanoDrop 2000c Spectrophotometer (Thermo Fisher Scientific, Waltham, MA, USA) was used to evaluate the quality of the RNAs. The RNAs were reverse-transcribed cDNA using the PrimeScript kit (Cat no. RR047A, Takara, Shiga, Japan) in accordance with the protocols of the manufacturer.

The target genes following *FRO1, FRO8, IRT1, NRT2.5, AMT1, MYB113*, and *DHS* were chosen from the DNA sequence of *A. thaliana* (Table 7). The CDS sequences of these target genes were found on the National Center for Biotechnology Information ‘https://www.ncbi.nlm.nih.gov/’ (accessed on 10 February 2022) and then compared with the draft genome sequence of *Petunia axillaris*: ‘https://solgenomics.net/organism/Petunia_axillaris/genome’ (accessed on 10 February 2022), before determining the gene expression levels. The primers are shown in Table 8. The gene expression was analyzed by SYBR Green Master Mix kit and quantitative real-time PCR (RT-PCR) was performed on a CFXconnect system (Bio-Rad, Hercules, CA, USA). The reaction system (10 µL) contained 1 µL of cDNA (100 µM), 1 µL of each primer (10 µM), 2 µL of ddH_2_O, and 5 µL of SYBR Green Master Mix. The melting temperature of gene-specific primers was designed to be 55 °C. The *PhActiin11* gene was selected as a reference and the data were analyzed using the 2^−∆∆Ct^ method. For each treatment, three biological replicates were employed.

### 4.11. Data Analysis

The analysis of variance (ANOVA) was used to evaluate significant differences between the treatments using the PROC ANOVA function in SAS (SAS, version 8, SAS Institute Inc., Cary, NC, USA). First, Fe pH was considered one-way, then the Duncan’s multiple range testing was conducted at a 5% probability level. Second, pH, Fe, and their interaction (A × B) were also analyzed. The analysis graphics were drawn with the OriginPro 9.0 software (OringinLab Co., Northampton, MA, USA).

## 5. Conclusions

The results of our study showed that the Fe source and medium pH affected the growth and development of *P. hybrida*. The Fe increased the leaf width, shoot fresh and dry weights, and root dry weight of *P. hybrida*. The contents of chlorophyll, carotenoids, and anthocyanins, as well as leaf color, regardless of the Fe source, were significantly affected by the pH, and the results decreased with increasing pH. The highest soluble protein content and activities of APX and CAT were observed in the Fe-EDTA under pH 5.70. In addition, the highest contents of NH_4_^+^ and NO_3_^−^ were measured in the FeSO_4_-4.7 and EDTA-5.7, respectively. When the Fe source was Fe-EDTA, pH 5.70 of the medium was the most beneficial for nutrient uptake of *P. hybrida*. The gene-expression results of *P. hyrida* revealed that the greatest relative gene expressions of *PhIRT1*, *PhFOR1*, *PhFRO8*, *PhNRT2.5*, and *PhDHS* were obtained with Fe-EDTA, pH 5.70. In the treatment of EDTA-5.7, the reduction and transport of chelated iron in *P. hybrida* leaves were enhanced, which also affected the transport of nitrate. In conclusion, when the medium pH was adjusted to 5.70, supplementation of chelated Fe-EDTA was more conducive to promoting the growth and development of, and absorption of mineral nutrients by, the plant, and expression of related genes in leaves. Therefore Fe-EDTA was more conducive to the growth and development of *P. hybrida* at pH 5.70.

## Figures and Tables

**Table 1:** Growth of *P. hybrida* after 10 days of culture in vitro.

Fe Source (A)	pH (B)	Leaf			Shoot			Root		
		Number	Length (mm)	Width (mm)	Length (mm)	Fresh Weight (mg)	Dry Weight (mg)	Length (mm)	Fresh Weight (mg)	Dry Weight (mg)
Control	4.70	9.3 ^z^ a ^y^	30.0 ab	6.0 c	4.0 bc	141.0 cd	13.7 f	49.0 b	57.5 c	2.3 c
	5.70	8.7 ab	21.3 c	7.8 a	6.3 a	236.7 a	17.1 de	49.5 b	70.0 b	3.0 c
	6.70	5.7 de	26.0 bc	7.7 ab	3.8 cd	128.5 d	17.3 de	21.5 e	18.3 e	2.6 c
FeSO_4_	4.70	9.0 ab	25.7 bc	5.7 c	4.7 bc	255.0 a	18.3 cd	42.7 c	55.3 c	4.2 b
	5.70	7.7 c	25.0 bc	8.7 a	3.7 cd	165.5 cd	20.5 bc	29.0 d	65.5 b	2.3 c
	6.70	6.3 cd	25.3 bc	7.8 a	3.5 cde	232.0 a	34.1 a	32.0 d	21.8 e	1.3 d
Fe-EDTA	4.70	8.7 ab	25.5 bc	7.3 abc	3.3 cde	253.0 a	21.4 bc	44.0 c	69.5 b	3.8 b
	5.70	8.7 ab	32.0 a	5.7 c	3.2 de	200.5 b	14.8 ef	55.0 a	118.6 a	6.1 a
	6.70	4.3 e	25.0 bc	8.5 a	2.8 e	130.0 d	17.2 de	32.5 d	41.0 d	2.5 c
*F*-test ^x^	A	NS	NS	NS	***	***	***	***	***	***
	B	***	NS	**	***	***	***	***	***	***
	A × B	NS	*	**	***	***	***	***	***	***

^z^ Values represent the mean of three replications. ^y^ Mean separation within columns by the Duncan’s multiple range test at 5% level. ^x^ NS, *, **, and ***: non-significant or significant at *p* ≤ 0.05, 0.01, or 0.001, respectively.

**Table 2:** The correlation analysis between the leaf color and plant pigment contents.

Fe Source (A)	pH (B)	Red Attribute	Green Attribute	Blue Attribute
Control	4.70	115.0 ^z^ d ^y^	126.5 cd	79.5 d
	5.70	153.0 c	165.0 b	132.5 ab
	6.70	209.5 a	210.7 a	136.5 a
FeSO_4_	4.70	123.5 d	178.7 b	113.7 bc
	5.70	172.0 b	139.5 c	112.5 bc
	6.70	221.0 a	210.3 a	130.5 abc
Fe-EDTA	4.70	115.0 d	114.0 d	76.5 d
	5.70	128.0 d	126.5 cd	85.5 d
	6.70	159.0 b	165.0 b	110.5 c
*F*-test ^x^	A	***	***	***
	B	***	***	***
	A × B	***	***	**

^z^ Values represent the mean of three replications. ^y^ Mean separation within columns by the Duncan’s multiple range test at 5% level. ^x^ ** and ***: significant at *p* ≤ 0.01 or 0.001, respectively.

**Table 3:** The correlation analysis between the leaf color and plant pigment contents.

Correlation	Red Attribute	Green Attribute	Blue Attribute	Total Chlorophyll	Carotenoid
**Green attribute**	0.944 ** ^z^	-	-	-	-
**Blue attribute**	0.735 **	0.819 **	-	-	-
**Total chlorophyll**	−0.717 **	−0.689 **	−0.405 *	-	-
**Carotenoid**	−0.790 **	−0.748 **	−0.483 *	0.974 **	-
**Anthocyanin**	−0.781 **	−0.737 **	−0.441 *	0.957 **	0.967 **

^z^ * and **: significant at the *p* ≤ 0.05 and 0.01 levels, respectively.

**Table 4:** Contents of macronutrients and micronutrients in leaves of *P. hybrida* after 10 days of culture in vitro.

Fe Source (A)	pH (B)	Macronutrients (mg·g^−1^ DW)					Micronutrients (mg·g^−1^ DW)			
		P	K	S	Ca	Mg	Cu	Mn	Zn	Fe
Control	4.70	102.7 ^z^ b ^y^	2029.2 b	103.3 c	459.6 b	109.0 c	5.93 a	0.83 b	2.01 a	7.00 b
	5.70	86.4 d	1500.9 d	567.1 a	295.7 de	88.0 e	3.25 cd	0.66 c	1.34 cd	5.93 cd
	6.70	99.8 bc	1000.5 e	306.9 b	309.8 d	118.7 b	5.02 ab	0.53 d	1.81 ab	3.56 e
FeSO_4_	4.70	91.8 cd	1834.4 c	117.5 c	362.8 c	92.4 de	4.12 bc	0.80 b	1.27 de	6.36 bc
	5.70	104.3 b	1003.0 e	98.7 c	282.9 de	70.3 f	3.91 bcd	0.64 c	0.97 f	3.68 e
	6.70	73.5 e	652.8 f	62.9 c	213.4 f	68.7 f	2.86 d	0.31 e	0.66 g	3.16 e
Fe-EDTA	4.70	105.6 b	1827.1 c	96.0 c	389.1 c	99.9 cd	4.62 b	0.80 b	1.10 def	5.07 d
	5.70	133.1 a	2286.9 a	150.2 c	686.6 a	153.9 a	4.41 bc	1.47 a	1.01 ef	9.09 a
	6.70	69.3 e	727.0 f	89.3 c	265.2 e	72.0 f	4.23 bc	0.36 e	1.58 bc	5.92 cd
*F*-test ^x^	A	***	***	***	***	***	***	**	***	***
	B	***	***	***	***	***	**	**	NS	***
	A × B	***	***	***	***	***	***	**	***	***

^z^ Values represent the mean of three replications. ^y^ Mean separation within columns by the Duncan’s multiple range test at 5% level. ^x^ NS, **, and ***: non-significant or significant at *p* ≤ 0.01, or 0.001, respectively.

**Table 5:** The correlation analysis between content of pigments and Fe in leaves of *P. hybrida* after 10 days of culture in vitro.

Correlation	Chlorophyll	Carotenoid	Anthocyanin
**Carotenoid**	0.976 ** ^z^	-	-
**Anthocyanin**	0.961 **	0.967 **	-
**Fe**	0.420 *	0.372	0.403 *

^z^ * and **: significant at the *p* ≤ 0.05 and 0.01 levels, respectively.

**Table 6:** The correlation analysis between growth parameters and nutrient contents in leaves of *P. hybrida* after 10 days of culture in vitro.

Correlation	Leaf Number	Shoot			Zn	Cu	Fe	Mn	Ca	Mg	K	P	S	NH_4_^+^
		Length	FW	DW										
**Length**	0.367 ^z^	-	-	-	-	-	-	-	-	-	-	-	-	-
**FW**	0.458 *	0.370	-	-	-	-	-	-	-	-	-	-	-	-
**DW**	−0.253	−0.147	0.416 *	-										
**Zn**	−0.040	0.083	0.556 **	−0.639 **	-	-	-	-	-	-	-	-	-	-
**Cu**	0.160	−0.247	−0.424 *	−0.523 **	0.667 **	-	-	-	-	-	-	-	-	-
**Fe**	0.178	−0.320	−0.271	−0.649 **	0.346	0.516 **	-	-	-	-	-	-	-	-
**Mn**	0.600 **	−0.030	0.178	−0.533 **	−0.048	0.259	0.731 **	-	-	-	-	-	-	-
**Ca**	0.496 **	−0.171	0.015	−0.553 **	0.058	0.354	0.845 **	0.960 **	-	-	-	-	-	-
**Mg**	0.336	−0.102	−0.081	−0.535 **	0.218	0.397 *	0.803 **	0.828 **	0.883 **	-	-	-	-	-
**K**	0.784 **	0.160	0.286	−0.566 **	0.136	0.398 *	0.655 **	0.881 **	0.848 **	0.714 **	-	-	-	-
**P**	0.524 **	−0.138	−0.009	−0.461 *	−0.024	0.319	0.634 **	0.883 **	0.851 **	0.813 **	0.724 **	-	-	-
**S**	0.112	0.748 **	0.115	−0.280	0.228	−0.259	−0.224	0.012	−0.083	0.130	0.030	−0.026	-	-
**NH_4_^+^**	0.317	0.157	0.292	−0.069	0.132	0.161	0.222	0.075	0.034	−0.019	0.299	−0.080	−0.272	-
**NO_3_^−^**	0.004	−0.164	0.352	0.276	−0.380	−0.190	0.119	0.295	0.317	0.485 *	0.157	0.296	0.154	−0.398 *

^z^ * and **: significant at the *p* ≤ 0.05 and 0.01 levels, respectively.

**Table 7:** The names and locus ID of target genes of *Arabidopsis thaliana* used in this study.

Gene Name	Abbreviation	Locus ID
Ferric reduction oxidase 1	*FRO1*	AT1GO1590
Ferric reduction oxidase 8	*FRO8*	AT5G50160
Iron-regulated transporter 1	*IRT1*	AT4G19690
Nitrate transporter 2.5	*NRT2.5*	AT1G12940
Ammonium transporter 1;1	*AMT1;1*	AT4G13510
Myb domain protein 113	*MYB113*	AT1G66370
Deoxyhypusine synthase	*DHS*	AT5G05920

**Table 8:** The list of primers used for gene expression quantification in *P. hybrida*.

Name	Gene Identifier	Forward (5′–3′)	Reverse (5′–3′)
*PhFRO1*	Peaxi162Scf41243	CGTCTCACAGGTCAAAACCC	TTGCTGGTTGGATTGATATTGTGG
*PhFRO8*	Peaxi162Scf00089	TCAGTCTTCTTGCAGGATTGAT	TGGAAGGAAAGATTCGAACAGA
*PhIRT1*	Peaxi162Scf00623	GCTGGGAATCATTGTTCACTC	CACCAAGTCCCATTCCTTCAAAC
*PhNRT2.5*	Peaxi162Scf00515	GGTGTTGAACTTACTGTGGACAA	CAAAAGTGAGGCCACAGGCA
*PhAMT1;1*	Peaxi162Scf00043	CCATAAACCAGCAATGCCACC	TGCAGGATCAGTTAGAGCCAA
*PhMYB113*	Peaxi162Scf00102	GGTGTGGAAAGAGTTGTAGGC	TGTCAGTTCGACCTGGTAATC
*PhDHS*	Peaxi162Scf00178	TGGCACCACCACGTATTTTTC	CTGTCCATGCTGGTTCAAGG
*PhActin11*	cn1159	TGCACTCCCACATGCTATCCT	TCAGCCGAAGTGGTGAAAGAG

## Data Availability

Data sharing is not applicable to this article.
